# A Comparison of Imaging Techniques to Monitor Tumor Growth and Cancer Progression in Living Animals

**DOI:** 10.1155/2011/321538

**Published:** 2011-11-10

**Authors:** Anne-Laure Puaux, Lai Chun Ong, Yi Jin, Irvin Teh, Michelle Hong, Pierce K. H. Chow, Xavier Golay, Jean-Pierre Abastado

**Affiliations:** ^1^Laboratory for Tumor Immunology, Singapore Immunology Network (SIgN), BMSI, A*STAR, Immunos Level 4, 8a Biomedical Grove, Singapore 138648; ^2^Singhealth Experimental Medicine Centre, Singapore General Hospital, Block 9 Level 3, Outram Road, Singapore 169608; ^3^Singapore Bioimaging Consortium, Biomedical Sciences Institutes, 11 Biopolis Way, No. 02-02 Helios, Singapore 138667; ^4^Office of Clinical Sciences, Duke-NUS Graduate Medical School 2, Jalan Bukit Merah, Singapore 169547; ^5^Department of General Surgery, Singapore General Hospital, Outram Road, Singapore 160608

## Abstract

*Introduction and Purpose*. Monitoring solid tumor growth and metastasis in small animals is important for cancer research. Noninvasive techniques make longitudinal studies possible, require fewer animals, and have greater statistical power. Such techniques include FDG positron emission tomography (FDG-PET), magnetic resonance imaging (MRI), and optical imaging, comprising bioluminescence imaging (BLI) and fluorescence imaging (FLI). This study compared the performance and usability of these methods in the context of mouse tumor studies. *Methods*. B16 tumor-bearing mice (*n* = 4 for each study) were used to compare practicality, performance for small tumor detection and tumor burden measurement. Using RETAAD mice, which develop spontaneous melanomas, we examined the performance of MRI (*n* = 6 mice) and FDG-PET (*n* = 10 mice) for tumor identification. *Results*. Overall, BLI and FLI were the most practical techniques tested. Both BLI and FDG-PET identified small nonpalpable tumors, whereas MRI and FLI only detected macroscopic, clinically evident tumors. FDG-PET and MRI performed well in the identification of tumors in terms of specificity, sensitivity, and positive predictive value. *Conclusion*. Each of the four methods has different strengths that must be understood before selecting them for use.

## 1. Introduction

Studies in living animals are critical to oncology research, and many experimental models have been exploited for drug development and basic studies [1, 2]. Fast-growing tumors can be generated in mice by orthotopic or ectopic implantation of tumor cell lines. However, models exhibiting spontaneous oncogenesis better mimic human disease therefore, oncogene-driven or chemically induced tumor models have come into use more recently [3, 4]. In both spontaneous and transplanted tumor models, the most common readouts are primary tumor growth and metastatic spread, but accurate measurement of these parameters is challenging. Unlike necropsy, noninvasive imaging techniques could offer an ideal solution as they allow measurement of tumor burden in the whole body without the need to sacrifice the animal. This makes longitudinal studies possible, simultaneously reducing the number of animals required and producing more robust data. These technologies are also sensitive and accurate enough to detect microscopic nodules, whose importance in human disease prognosis is increasingly recognized [5, 6]. 

Several imaging techniques have recently become available for small animals [7]. These include 2-deoxy-2-[18F]fluoro-D-glucose positron emission tomography (FDG-PET) [8], T2-weighted magnetic resonance imaging (T2W-MRI) [9], and optical imaging, encompassing bioluminescence imaging (BLI) [10] and fluorescence imaging (FLI) [11]. Both FDG-PET and T2W-MRI are used clinically in humans, whereas optical imaging is specifically used for research and preclinical studies. While each method has its own advantages, a detailed side-by-side comparison of their use for tumor imaging has yet to be carried out.

The purpose of the present study is to compare practicality and performance of these four imaging techniques in the context of mouse tumor studies. Specifically, we assessed four different parameters, namely, practicality, performance for small tumor detection, performance for tumor burden measurement, and performance for tumor identification. The first two parameters were comparatively assessed across all four imaging technologies. The performance for tumor burden measurement was conducted specifically for optical methods, since they are well adapted for this purpose. Conversely, the performance for tumor identification was compared only between MRI and FDG-PET, since optical methods cannot be applied for this purpose in current tumor models.

We used two murine melanoma models to conduct the proposed comparisons. The B16 transplanted tumor model is well-defined and offers a high level of flexibility [2]. B16 cells can be modified to express the transgenes required for detection by optical imaging, followed by injection of these cells into the animal by different routes to produce either subcutaneous or pulmonary lesions [2]. Tumor onset is predictable, so nodules can be tracked from their microscopic stage, making this model ideal to assess the practicality and performance of each technique for small tumor detection and tumor burden measurement. The second model is the RETAAD mouse which spontaneously develops melanoma tumors and metastases [3]. In contrast to transplanted tumor models, RETAAD tumors may arise at any location in the skin (cutaneous melanoma tumors) and internal organs (visceral metastases). This model is, therefore, particularly suited for the assessment of the performance of imaging techniques in tumor identification, as it makes it possible to calculate the specificity, the sensitivity, and the positive predictive value for tumor detection. Tumors in spontaneous models usually do not express reporter genes and are, therefore, not suited for optical imaging technologies. Therefore, we have used this model to compare FDG-PET and T2W-MRI.

## 2. Materials and Methods

### 2.1. Cell Lines

Stably transfected B16 cells were used for detection by optical imaging techniques. B16-F10-luc cells (Xenogen, Alameda, Calif, USA) express firefly luciferase (sequence from pGL3, Promega) under the control of the SV40 promoter. B16-F10-RFP cells express DsRed2 under the control of the CMV promoter [11, 12].

### 2.2. Animals

All studies were approved by the Institutional Animal Care and Use Committee of the Biological Resource Center and of Singhealth. C57Bl/6 mice were inoculated subcutaneously or intravenously with 10^5^ B16-F10-luc or B16-F10-RFP cells. RETAAD mice were generated as previously described [3].

### 2.3. Clinical Examination

B16-injected mice were examined by palpation at the site of injection. Two perpendicular diameters (*d*_1_ ≤ *d*_2_) of the tumor were then measured using caliper, and were used to calculate the tumor volume (V) (*V* = 4/3 · *π* · *d*_1_^2^ · *d*_2_/8).

### 2.4. Necropsy Analysis

Prior to necropsy, the investigator was unaware of the results of the imaging scans, rendering the two analyses independent. Mice injected subcutaneously with B16 cells were examined at the injection site. Animals injected intravenously with B16 cells were examined for their lungs and peritoneal cavity as described [2]. RETAAD mice develop tumors spontaneously with widespread metastases, so they were subjected to more extensive necropsy. For each mouse, a necropsy diagram was filled to document the location, size, and morphology of nodules.

### 2.5. 18-Fluoro-Deoxy-Glucose-Positron Emission Tomography Scan (FDG-PET)

Four mice subcutaneously injected with B16 cells were used to determine the smallest detectable tumor. Ten RETAAD mice were used to assess performance in tumor identification. 

After fasting overnight, mice were prewarmed to 37°C, and approximately 5.5 MBq of FDG (0.6 mM) (Department of Nuclear Medicine, Singapore General Hospital) was administered intraperitoneally [14]. Mice were then maintained at 37°C throughout the one-hour uptake period [14]. Micro-PET imaging was performed using a R4 microPET scanner (Concordes Microsystems Inc.) with a ring diameter of 26 cm, 7.8 cm axial field of view and an average intrinsic spatial resolution of 1.75 mm. Under isoflurane anesthesia, mice were subjected to 15 minutes of acquisition. For image reconstruction, an energy window of 350–700 keV and a coincidence timing window of 6ns were used. Two-dimensional histograms by Fourier rebinning and image reconstruction by filtered backprojection were used. The image data were corrected for nonuniformity of the scanner response, dead time count losses, and physical decay to the time of injection. No correction was applied for attenuation, scatter, or partial-volume averaging, as these parameters are not critical for mouse models [15].

In the reconstructed images, tumors were identified as regions of high uptake in study animals that were absent from images of control mice. To allow quantitative image analysis, regions of interest (ROI) were manually drawn over areas of high uptake. Within these regions, counting rates were converted to standardized uptake values (SUVs) using a system calibration factor derived from the imaging of a mouse-size water-equivalent phantom containing ^18^F.

### 2.6. T2-Weighted Magnetic Resonance Imaging (T2W-MRI)

Four mice subcutaneously injected with B16 cells were used to determine the smallest detectable tumor. Six RETAAD mice were used to assess performance in tumor identification.

Data were acquired at the Singapore Bioimaging Consortium on a 9.4T MRI scanner (Varian, Palo Alto, Calif, USA) using a transmit-receive volume RF coil. A multislice 2D fast spin echo with periodically rotated parallel lines with enhanced reconstruction (PROPELLER) pulse sequence [16] was used to give high image quality and robustness to motion. Scans were performed for the brain and abdominal regions under isoflurane anesthesia. Brain scans were conducted using the following parameters: repetition time (TR) = 4000 ms; effective echo time (TE) = 51 ms; echo spacing (ESP) = 6.4 ms; echo train length (ETL) = 16; field-of-view = 25.6 × 25.6 mm; blade matrix = 256 × 16; number of blades = 32; reconstructed matrix = 256 × 256; slice thickness = 1 mm; slice gap = 0.5 mm; slices = 5; averages = 1; orientation = axial; readout bandwidth = 208 kHz and acquisition time = 2 min 16 s. For the abdomen, several changes were made to accommodate the shorter T2 so that, TE = 20 ms; ESP = 5.0 ms; ETL = 8; blade matrix = 128 × 8; reconstructed matrix = 128 × 128; slice gap = 0.2 mm. The resulting data were used to reconstruct images as described [17]. Tumors were identified as highly contrasted masses or nodules that were present in study animals but absent from control mice.

### 2.7. Bioluminescence Imaging (BLI)

For all BLI experiments, the B16-luc cell line was used. Studies to determine the smallest detectable tumors, accuracy for tumor burden measurement, and tissue attenuation used four, four, and six mice, respectively, each subcutaneously injected with B16 cells. To further demonstrate the possibility to use BLI for accurate followup of tumor growth (as shown in Figure 5), 4 unshaved mice were injected subcutaneously with B16 cells and another 4 were injected intravenously. For other experiments, mice were shaved as indicated in the figure legends. 

15–25 minutes before imaging, mice were injected intraperitoneally with 200 *μ*L of D-luciferin (15 mg/mL in PBS) as described [10] and then anesthetized using isoflurane. For *in vitro* imaging, cells were plated in PBS in flat-bottomed 96 well plates before D-luciferin was added to a final concentration of 1.5 mg/mL. Immediately after necropsy, some tumors were excised and imaged *ex vivo* on tissue culture plates. These plates were scanned for 5 to 40 seconds, whereas mice were scanned for 30 to 60 seconds using the IVIS Spectrum photon-counting device optical imaging system (Xenogen, Alameda, Calif, USA). Regions of interest were drawn and quantified using the Living Image software version 2.5. Bioluminescence signal was reported as total light emission within the region of interest (photon/s). Specific signal was calculated as the ratio of bioluminescent signal in the region of interest to the bioluminescent signal in a background region containing no cells or tumors. A signal was defined as positive when it was greater than the sum of the mean background signal plus 2 standard deviations of the background signal.

### 2.8. Fluorescence Imaging (FLI)

For all FLI experiments, the B16-RFP cell line was used. Studies to determine the smallest detectable tumors, accuracy for tumor burden measurement, and tissue attenuation used four, four, and six mice, respectively, each subcutaneously injected with B16 cells. 

For* in vivo* imaging, animals were anesthetized using isoflurane and some mice were shaved as indicated in the figure legends. For *in vitro* imaging, cells were plated in PBS in flat-bottomed 96-well plates. Immediately after *in vivo* imaging, some tumors were excised at necropsy and imaged *ex vivo* on tissue culture plates. Plates or mice were scanned for 0.1 to 1 seconds using the IVIS Spectrum photon-counting device optical imaging system (Xenogen, Alameda, CA) with filters for red fluorescence (excitation 535 nm, emission 600 nm) and background fluorescence (excitation 465 nm, emission 600 nm). Regions of interest were drawn and quantified using Living Image software version 2.5. Fluorescence background was subtracted according to the manufacturers' instructions. Fluorescence signal was reported as light conversion efficiency. Specific signal was reported as the ratio of the fluorescence signal in the region of interest to the fluorescence signal in a background region containing no cells or tumors. A signal was defined as positive when it was greater than the sum of the mean background signal plus 2 standard deviations of the background signal.

### 2.9. Statistical Analysis

BLI and FLI specific signals (signal-to-noise ratio) for single time point experiments were compared using the Mann-Whitney test. 

Tumor growth curves were compared using a nonparametric test according to [13].

Tumor optical imaging signal and tumor volume were compared using Spearmann correlation. 

The specificity was defined as (number of sites where no tumor was found)/(total number of sites without tumor confirmed at necropsy), where the total number of possible sites for tumor growth in each RETAAD mouse was 14 (cheeks, neck, genitals, flanks, forelimbs, hind limbs, and peritoneum, each time on the left or right side).

The positive predictive value was defined as (number of tumors detected by imaging and confirmed at necropsy)/(total number of tumors detected by imaging).

The sensitivity for tumor identification was defined as follows: (number of tumors detected by imaging and confirmed at necropsy)/(total number of tumors observed at necropsy).

## 3. Results

### 3.1. Practicality

Three parameters were taken into account: animal preparation, time for analysis, and ease of access to the technology (Table 1).

All techniques required anesthesia of the animal by isoflurane inhalation, taking approximately 4 minutes per mouse. The additional tracer injection and preincubation time for FDG-PET resulted in at least 3-fold longer preparation time per animal compared to the other techniques. Shaving requires 10 minutes per animal, and while there has been debate on whether it can be omitted for optical imaging [11], we found it to be dispensable for BLI (see below).

We next compared the time needed for image acquisition and analysis. Because devices differ, we selected widely used platforms for comparison: IVIS Spectrum (Xenogen) for optical imaging, R4 microPET (Concordes Microsystem) for FDG-PET, and 9.4T MRI (Varian) for T2W-MRI. The main differences in practicality between technologies were highlighted by the ease of scale-up to larger groups of animals. For example, the IVIS Spectrum allows parallel imaging of up to 5 mice, taking as little as 2 minutes to complete all the scans. In contrast, both FDG-PET and T2W-MRI can typically image only one mouse at a time (maximum 2 in some settings), taking at least 15 minutes per scan. Overall, this meant that optical scanning of 10 mice could be completed in 1-2 hours, whereas FDG-PET or T2W-MRI would take 13–30 hours. 

The last parameter considered was cost and availability. Each technique has low reagent and consumable costs of around five US dollars per scan per animal. FDG can usually be obtained as surplus material from nuclear medicine departments or comes at a low cost compared to other PET reagents. Equipment for optical imaging is accessible in many research institutes and costs less than five hundred thousand US dollars, with the PET scanner costing around six hundred thousand US dollars. MRI scanners are most expensive, costing one to two million US dollars. 

Overall BLI and FLI are the most practical techniques and are particularly suitable for large studies requiring high throughput imaging.

### 3.2. Determination of the Smallest Detectable Tumor

The B16 melanoma model allowed us to assess the smallest tumors that could be accurately detected by the various techniques. Four mice per technique were injected subcutaneously with 10^5^ B16 cells, resulting in tumor growth at the injection site [2]. These tumors were clinically undetectable up to day 10 after injection, but were evident at necropsy. After day 10, tumors become macroscopic (2 mm diameter and above) and were measured in living animal with a caliper.

**Figure 1:**
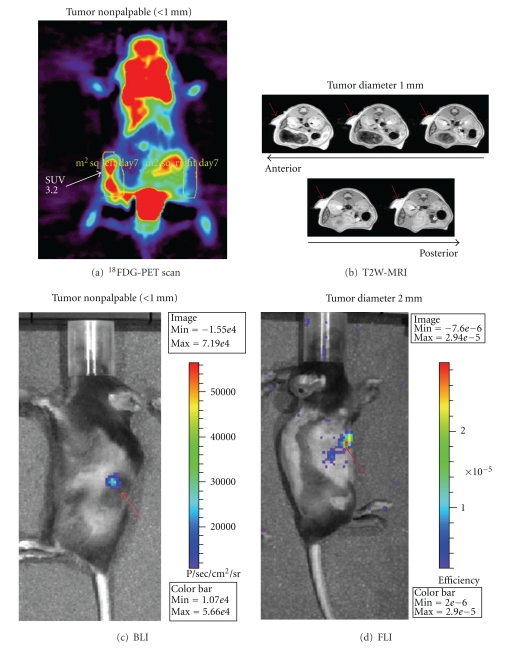
Detection of tumors by *in vivo *imaging. B16 melanoma cells were subcutaneously injected into shaved C57Bl/6 mice (*n* = 4 mice for each technique). Mice were repeatedly imaged by: (a) FDG-PET, (b) T2W-MRI, (c) BLI, and (d) FLI. For each technique, a representative mouse is shown, and the smallest detected tumor is reported. Arrows indicate tumors. SUV, standardized uptake value.

BLI and FDG-PET detected nonpalpable tumors (<1 mm), whereas the smallest tumors detected by T2W-MRI and FLI were 1 mm and 2 mm diameter, respectively (Figure 1). BLI detected microscopic tumors as early as 1 day after subcutaneous injection (Figure 1(c)) when the nodules were too small to be detected even at necropsy. While this means that their presence could not be confirmed either visually or histologically, these tumors were actively growing, increasing their BLI signal and could indeed be identified at necropsy by day 2 (data not shown). Overall, BLI and FDG-PET are applicable for *in vivo* detection of microscopic tumors, whereas T2W-MRI and FLI are only applicable to palpable tumors.

**Figure 2:**
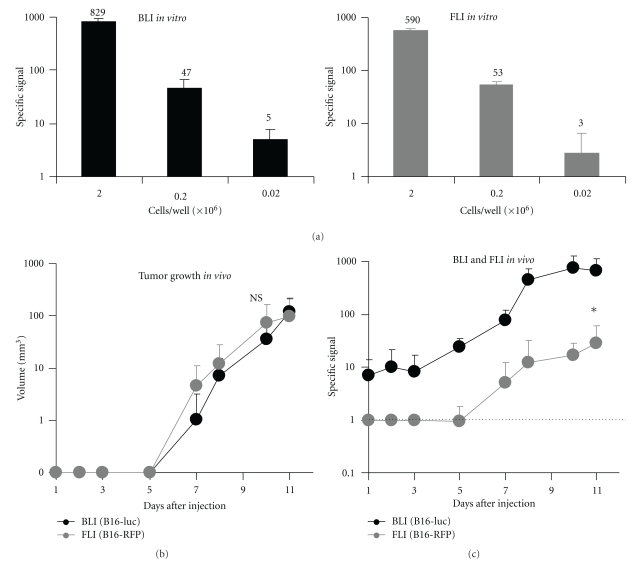
Tumor growth monitored *in vivo* by optical imaging. (a) Signal to background ratio is similar for BLI (B16-luc, left) and FLI (B16-RFP, right) *in vitro*. Results are shown as the mean and SD of 4 replicate wells from 2 independent experiments. (b) B16-luc and B16-RFP tumors grow equally *in vivo*. Following subcutaneous injection of cell lines into mice (*n* = 4 mice for each cell line), tumor volume was calculated from caliper measurements. Four tumors were measured for each cell line. NS, no significant difference between B16-luc and B16-RFP (*P* > 0.05, test according to [13]). (c) BLI is more sensitive for tumor detection *in vivo*. B16-luc or B16-RFP cells were injected subcutaneously. Mice (*n* = 4 mice each for BLI and FLI) were shaved and imaged. Four tumors were measured for each cell line. The dotted line represents the detection threshold calculated based on control tumors not expressing the relevant reporter gene. *, significant difference between B16-luc and B16-RFP (*P* < 0.05, test according to [13]).

Both optical imaging techniques can be used to follow transplanted tumor growth. To carry out a detailed comparison, we selected two B16-luc and B16-RFP clones showing equivalent performance for *in vitro* imaging (Figure 2(a)) with similar growth rates *in vitro* (not shown) and *in vivo* (Figure 2(b)). At day 1, 2, 3 and 5 after injection, only BLI detected nonpalpable tumors (Figure 2(c)). At further time points, the tumor-specific signal detected by BLI was significantly greater than that seen by FLI for tumors of equivalent size (Figure 2(c)). Both techniques may, therefore, be used to follow small macroscopic tumors, but only BLI provides data at the microscopic stage.

### 3.3. Effects of Tissue Attenuation on Small Tumor Detection by Optical Imaging

We investigated the higher sensitivity observed for BLI compared to FLI when performed *in vivo*. One hypothesis is that tissue attenuation affects FLI more than BLI. Attenuation occurs when tissues around the tumor absorb some of the imaging excitation and emission signal, autofluoresce, leading to a reduction in the signal to noise ratio.

**Figure 3:**
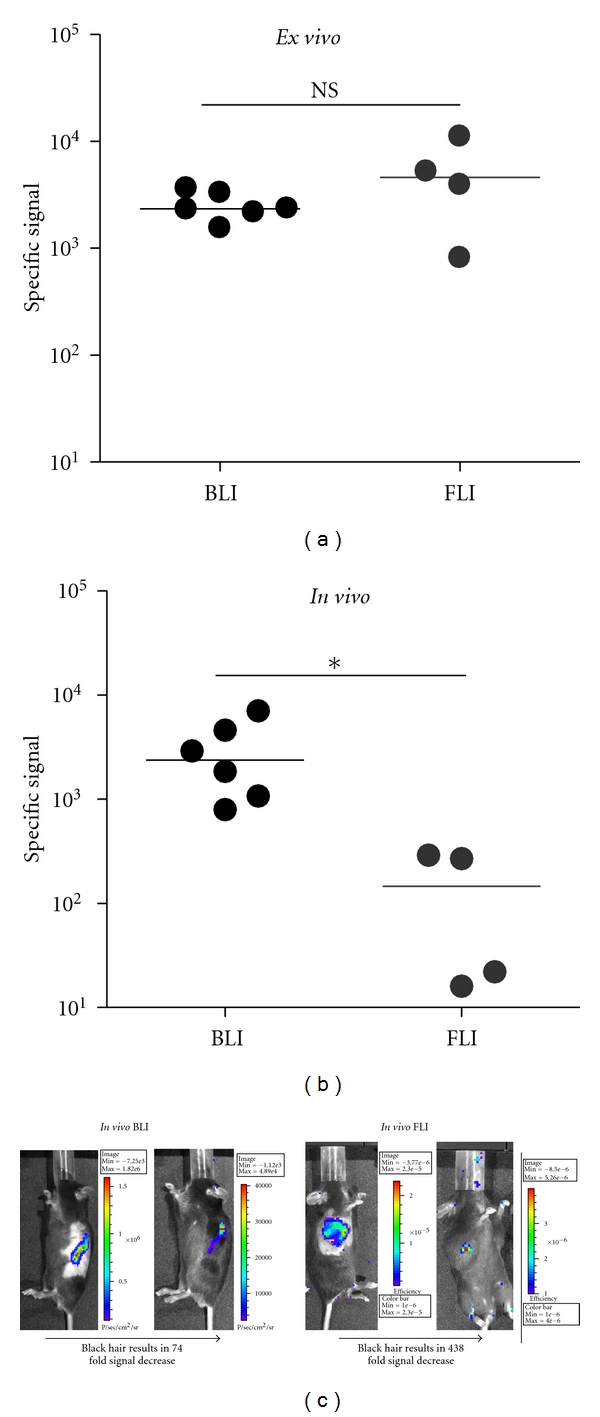
Mouse tissue attenuation is stronger for FLI than BLI. (a) B16-luc and B16-RFP tumors have similar specific signal *ex vivo*. Excised tumors from a total of 5 mice (*n* = 3 for BLI and *n* = 2 for FLI) were subjected to imaging. Data for individual tumors (*n* = 6 for BLI and *n* = 4 for FLI) and the median are shown. NS, no significant difference (*P* = 0.11, Mann-Whitney test). (b) Optical signal is strongly decreased *in vivo* for FLI on shaved mice. Tumors were scanned *in vivo* before excision. Data for individual tumors (*n* = 6 for BLI and *n* = 4 for FLI) and the median are shown. The tumors are the same as those described in (a). *, significant difference (*P* = 0.027, Mann-Whitney test). (c) Mouse hair strongly decreases the optical signal for FLI. B16-luc and B16-RFP tumors were imaged *in vivo* before and after shaving. A representative mouse for each technique (*n* = 3 and *n* = 2 mice analyzed for BLI and FLI, resp.) is shown.

To test this hypothesis, we measured signal reduction by comparing *ex vivo* (Figure 3(a)) and *in vivo* (Figure 3(b)) signals after tumor excision. The signal measured *in vivo* on shaved mice was reduced 3-fold for BLI and 14-fold for FLI compared to the signal of tumors *ex vivo* after excision (Figure 3(b)), confirming the hypothesis that FLI is more prone to tissue attenuation. To address the contribution of mouse hair to further signal attenuation, we imaged tumor-bearing mice before and after shaving. Again, the FLI signal (over than 400 fold reduced) was more prone to attenuation than the BLI signal (70 fold reduction) (Figure 3(c)).

In summary, tissue and hair surrounding the tumor significantly reduced the ability of FLI to detect small tumors *in vivo*. This tissue attenuation effect is higher for FLI than for BLI.

### 3.4. Accuracy of Optical Imaging for Measuring Tumor Burden *In Vivo*

**Figure 4:**
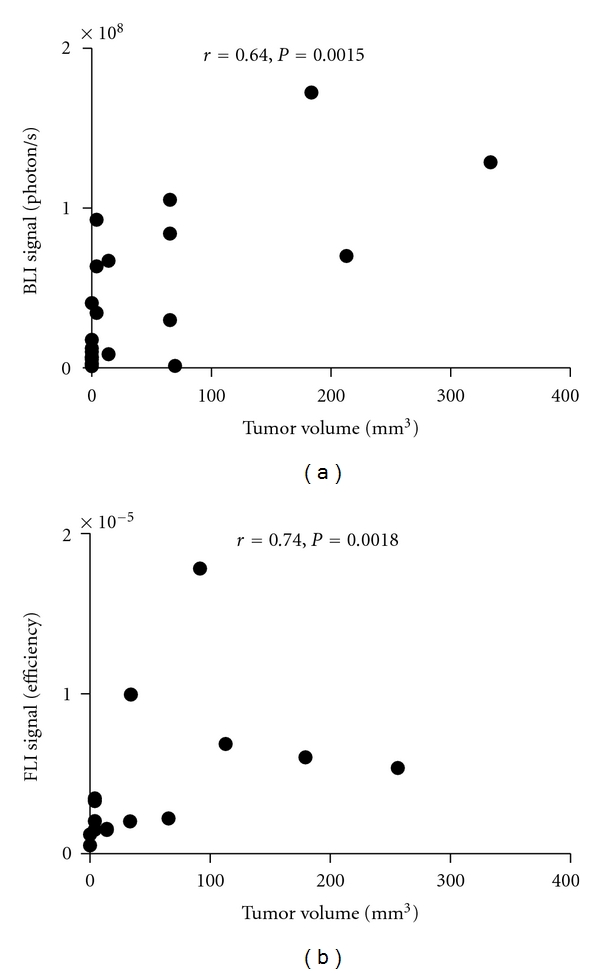
Tumor optical signal correlates with tumor volume calculated from caliper measurement. Data shown are BLI detection of luciferase-expressing tumors (*n* = 22, left) and FLI detection of RFP-expressing tumors (*n* = 15, right) in shaved mice. Only tumors displaying an optical signal above the background are shown. Tumors were derived from 4 mice each for BLI and for FLI. Tumors were imaged and tumor sizes were measured at various time points. Optical signal and tumor volume were compared using Spearman correlation.

Traditionally, caliper measurements are used to calculate tumor volume. We compared tumor volumes estimated *in vivo* by BLI and FLI, to those calculated by caliper measurements and found a good correlation between these two techniques (Figure 4). Therefore, optical imaging is appropriate to assess tumor burden.

**Figure 5:**
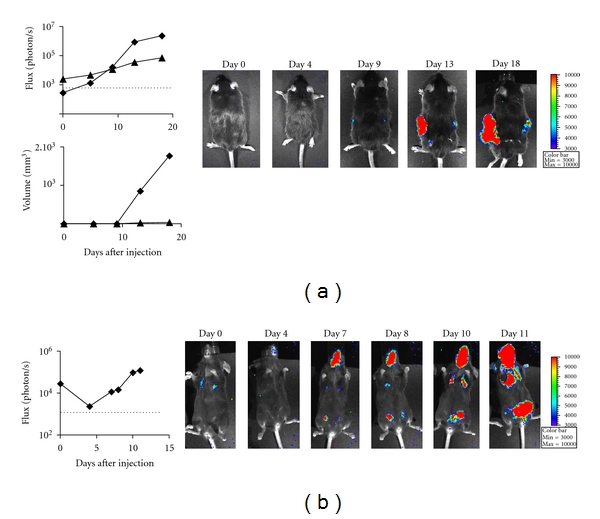
BLI detects both superficial and internal tumors on unshaved mice. (a) Followup of tumor growth *in vivo* after subcutaneous injection of B16-luc, using *in vivo* imaging and caliper measurement. A representative mouse of 4 mice is shown. The dotted line represents the detection threshold calculated based on control areas not expressing the relevant reporter gene. (b) Followup of tumor growth using *in vivo* imaging after intravenous injection of B16-luc. A representative mouse of 4 mice is shown. The dotted line represents the detection threshold calculated based on control areas not expressing the relevant reporter gene.

BLI has demonstrated an ability to detect microscopic tumors and to estimate tumor volumes *in vivo* with good accuracy. To further explore the power of the technique we injected unshaved mice either subcutaneously or intravenously with B16-luc cells and imaged them repeatedly. As expected, BLI detected subcutaneous tumors earlier than clinical examination (Figure 5(a)). Moreover, the bioluminescent signal follows a characteristic Gompertzian curve as expected for tumor growth [18], therefore more accurately reflecting the biology of the tumor compared to caliper measurements. BLI detected a signal following intravenous injection of B16-luc only after a few minutes, which likely reflects the initial trapping of the injected cells in the lung. By day 4 most of these cells were cleared and the BLI signal dropped, only to increase again as tumor growth occurred in the lungs, peritoneal cavity and at the point of injection (Figure 5(b)). These tumors were confirmed by necropsy (data not shown). BLI can, therefore, be used in shaved or unshaved mice for quantitative followup of tumor growth at both cutaneous and internal sites.

### 3.5. Specificity, Sensitivity, and Predictive Value of FDG-PET and T2W-MRI in a Spontaneous Tumor Model

Spontaneous or carcinogen-induced tumor models are increasingly used for cancer research. In these animals, a variable number of tumors arise in a range of locations over a less predictable time course. Macroscopic tumors are then assessed at necropsy, which is considered ground truth. The need to sacrifice the animal for information is a disadvantage of such models, but to replace necropsy by *in vivo* imaging, three criteria must be met. Firstly, the technique must correctly predict the absence of tumors at normal sites and for nontumor bearing mice (specificity). Secondly, the technique must identify tumors accurately, with a low rate of false positives (high predictive value). Thirdly, the technique must be sufficiently sensitive to detect all the tumors that necropsy currently does. We compared the specificity, predictive value and sensitivity of FDG-PET and T2W-MRI in the RETAAD spontaneous melanoma model. During the course of disease, these mice develop tumors of various sizes in wide-ranging anatomical locations, making the model an ideal test for the performance of these techniques. 

**Figure 6:**
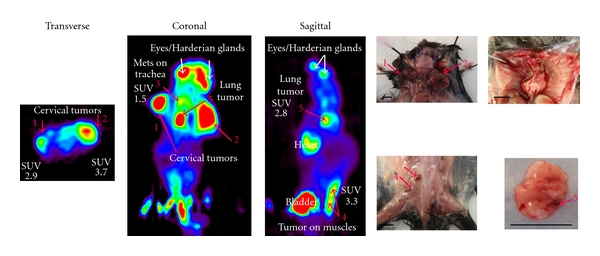
Identification of spontaneous tumors *in vivo* by FDG-PET. A RETAAD mouse (representative of 10 mice) was analyzed by FDG-PET, followed by necropsy. Tumors are indicated by red arrows and the numbers show the tumors identified both by FDG-PET and by necropsy. Standardized Uptake Value (SUV) is calculated as defined in Materials and Methods. Bar scale, 5 mm.

A representative FDG-PET scan is shown in Figure 6. Some background is evident in the bladder, heart, and eye regions, but this was expected due to the excretion and circulation of the probe, and the presence of the Harderian glands. The same effect was seen in control mice, and these regions were accordingly excluded from analysis. A total of 10 RETAAD mice and 4 control mice were independently analyzed by FDG-PET and necropsy. Of the 28 tumors identified by FDG-PET, 24 were confirmed by necropsy or histology, making the positive predictive value of the FDG-PET 86%. The 4 tumors that were not confirmed at necropsy were embedded in the muscles of the back and the limbs, sites for which histological analysis could not be carried out. An additional ten tumors were found at necropsy but not by FDG-PET, most likely because of low metabolic activity. Overall, the sensitivity of FDG-PET was 70%. For specificity, only 4 tumors were predicted by FDG-PET but could not be confirmed at necropsy or histology. Taking into account 14 possible sites for tumor growth for each of 14 mice analyzed, the specificity of FDG-PET was 98%.

**Figure 7:**
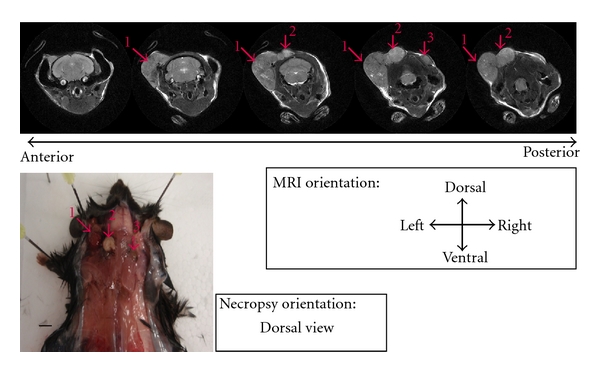
Identification of spontaneous tumors by T2W-MRI. A RETAAD mouse (representative of 6 mice) was analyzed by T2W-MRI, followed by necropsy. Tumors are indicated by red arrows and the numbers show the tumors identified both by T2W-MRI and by necropsy. Bar scale, 5 mm.

Figure 7 shows a typical T2W-MRI scan of a tumor-bearing RETAAD mouse. A total of 6 RETAAD mice and 4 control mice were independently analyzed by T2W-MRI and necropsy. Twenty-two tumors were identified by T2W-MRI, of which 21 were confirmed at necropsy, making the predictive value of T2W-MRI 95%. Five small tumors (4 out of 5 were <1 mm diameter) were observed at necropsy but not detected by imaging. The sensitivity of T2W-MRI was, therefore, 81%. For specificity, only one tumor was predicted by MRI and not confirmed at necropsy. Taking into account 14 possible sites for tumor growth for each of 10 mice analyzed, the specificity of T2W-MRI was 99%. Results are summarized in Table 2.

Overall, both FDG-PET and T2W-MRI allow precise 3D visualization of tumors with good specificity, sensitivity, and accuracy and are, therefore, highly recommended for any study aiming at identifying tumors. Interestingly, FDG-PET shows slightly lower sensitivity for tumor detection than MRI, probably due to the fact that some tumors lack the minimal metabolic activity required for detection. Therefore, FDG-PET would be the preferred choice if the assessment of metabolic activity is desired; otherwise, MRI is recommended (Table 2).

## 4. Discussion

### 4.1. BLI Versus FLI for Whole Body Tumor Imaging

To compare BLI and FLI *in vivo*, we used tumors originating from two different B16 cell lines expressing firefly luciferase and DsRed2, respectively. The two prototypical reporter genes have been chosen among the most commonly used and most efficient markers at the time of writing. Results obtained might change in the future when new reporters are developed. With current reporter genes, both BLI and FLI detected their respective cell lines equally well *in vitro*, but when the cells were injected into mice and allowed to form tumors, only BLI was able to image microscopic nodules. We showed that this difference was due to the tissues surrounding the tumor during *in vivo* imaging, a phenomenon known as tissue attenuation. This is especially relevant for FLI as the tissue can absorb and scatter fluorescent light at both the excitation and emission level. For BLI, there is no excitation involved so only the emission is subject to attenuation. These experiments used subcutaneous tumors, but for internal tumors the differences between the techniques could only be expected to be magnified due to the increased optical path through the tissues. 

Despite FLI being less sensitive than BLI with the instrumentation we used, it has been used successfully for whole-body imaging in other studies [11], even with very low cell numbers [19]. Such differences likely relate to variations in experimental protocol (e.g., see [20]). In addition, FLI has numerous applications beyond the scope of this study. For example, labeled proteins have enabled fluorescent imaging of tumor cell mobility, invasion and angiogenesis (reviewed in [12]). Hirakawa et al. successfully used FLI to monitor the dissemination of very small numbers of GFP-labeled skin tumor cells to the proximal lymph nodes of mice *in vivo* [21]. Importantly, FLI is so far the only imaging technology to give single-cell resolution [22] or even subcellular resolution *in vivo* [23, 24]. 

While each technique tested is state of the art, imaging technologies are constantly being improved. For example, optical techniques are being modified to permit three-dimensional reconstruction of the bioluminescent source and tumor localization [25]. It is even becoming possible to combine imaging modalities using multiple fusion reporter genes within the same animal (see, e.g., [26]).

### 4.2. Method of Choice for Whole-Body Tumor Imaging

Table 3 summarizes the main features of the imaging methods, with their primary advantages and disadvantages. 

Estimating the real cost of the different technologies is difficult, and largely depends on equipment availability. However, on the basis of equipment costs, operating expenses, and the level of training required, optical imaging is normally less costly than T2W-MRI and FDG-PET. 

While the design of the current study did not involve the comparison of all techniques using a single tumor-bearing animal, the B16 model is reproducible enough to carry out a fair comparison. By using groups of at least 4 mice to perform statistical analyses, we were able to detect some major differences between the various imaging modalities investigated. Further studies that include larger number of animals could be performed in order to detect even more subtle differences between these various imaging techniques.

Optical imaging is limited by its requirement for tumors to express a reporter gene. This is achievable in transplanted tumor models, but more challenging in spontaneous models. In fact, doing so requires generation of transgenic mice expressing the reporter gene in the cell lineage of interest, followed by either carcinogen treatment or intercrossing with an oncogene driven transgenic mouse line. This has been achieved in some cases. For example, Vooijs et al. expressed luciferase under a pituitary gland-specific promoter in a model of spontaneous pituitary cancer [27]. Similarly, Lyons et al. constructed an oncogen-driven prostate cancer model with luciferase expression in the prostate [28]. Consistent with our findings, both authors successfully monitored tumor growth *in vivo* using bioluminescence. Therefore, optical imaging is a valid strategy, but it is time consuming for spontaneous tumor models.

In contrast to optical imaging techniques, T2W-MRI and FDG-PET can be applied to any tumor-bearing mice, including spontaneous tumor models. Using a B16 mouse tumor model, we showed that T2W-MRI and FDG-PET scans allow early detection of tumors and exhibit good sensitivity and positive predictive value when compared to necropsy. Results obtained with PET scanning are related to tumor metabolism and glucose uptake by the tumors; hence, they could vary from one tumor cell line to the other. However, published data have shown that PET sensitivity is high in other tumor models (see,e.g., [8]). T2W-MRI performed slightly better, presumably because its basis is anatomical rather than requiring tumor metabolism, as in the case of FDG-PET. Metabolic rate assessment is a key parameter when measuring treatment success. Indeed, in treated cancer patients, some responsive tumors simply lose their metabolic activity while the tumor mass is unchanged. This typically translates into a tumor mass anatomically identified by MRI or CT scan but FDG-PET negative. In addition, MRI and FDG-PET are less affected than optical imaging by attenuation due to the depth of the tumor, and both have the significant advantage of providing precise locations of even small nodules. As MRI and FDG-PET are used in the clinical setting, their application in preclinical research may help translate basic findings into clinical studies. In this context, if whole-body metabolic imaging is required, FDG-PET is the best option for longitudinal followup of tumor burden and can be combined with CT which we did not address here. T2W-MRI is better used for specific body sections or to monitor the development of a particular tumor over time, as it may provide contrast and anatomical information related to location, volume, vascularization, and invasion. Further improvements in MRI technologies are increasing its applications. For example, diffusion-weighted imaging was successfully used to detect glioma tumors in rats [9], and more recently used for whole-body imaging [29].

## 5. Conclusion

Overall, this study has highlighted how various imaging techniques can be best used in different types of tumor models or to assess particular readouts. Our findings are also likely to be easily applicable to other species including rats. Optical imaging technologies are accessible, accurate, and specific. BLI in particular offers fast, sensitive whole-body tumor imaging, even detecting microscopic tumors. BLI could replace traditional caliper measurements, as it is able and well suited to determine tumor burden in longitudinal studies. However, the main disadvantage of optical techniques is the requirement for tumor cells to express a reporter gene. This has so far largely limited the use of BLI and FLI to transplanted tumor models. However, with time and resources, spontaneous tumor models that also express reporter genes will become increasingly available. 

In the meantime, however, this means that nonoptical methods are preferable for tumor detection in spontaneous models. Both available techniques have specific advantages and disadvantages; T2W-MRI accurately reflects tumor volume and morphology, but it is more time consuming, whereas FDG-PET uniquely measures metabolic activity. In summary, each technique represents a valuable tool to study tumor-bearing animals, but the careful selection of the most appropriate method will be critical to maximize the benefit of their use.

## Figures and Tables

**Table 1:** The process of analyzing tumor burden in whole mice.

	FDG-PET	T2W-MRI	BLI	FLI
Operating costs	Around five US dollars per time point per animal for all techniques			
Equipment costs	~600,000 US dollars	1 to 2 million US dollars	<500,000 US dollars	<500,000 US dollars
Mouse preparation	Anesthesia, tracer injection, incubation time and positioning	Anesthesia, set up of monitoring, prescan for positioning	Anesthesia, substrate injection, incubation time, shaving (optional), and positioning	Anesthesia, shaving and positioning
Mouse preparation time	1 h 30 min	30 min	20 min	5 min
Scanning time	15 min/3D scan	30 min/2D multislice scan/area (2 areas scanned per mouse)	1 s–2 min/picture 1 to 10 pictures/scan	1 s–30 s/picture 1 to 10 pictures/scan
Data analysis	Requires expertise	Requires expertise	Straightforward	Straightforward
Data analysis time	1 h	1 h 30 min	20 min	20 min
Total time 1 animal	3 h	3 h	1 h	30 min
Total time 10 animals	13 h	30 h	2 h	1 h

**Table 2:** MRI and FDG-PET performance for tumor identification assessed using RET-AAD mice.

	FDG-PET	T2W-MRI
Number of mice	10 RET-AAD mice, 4 control mice	6 RET-AAD mice, 4 control mice
Number of tumors (necropsy)	34	26
Number of tumors (imaging)	28	22
Positive predictive value	86%	95%
Sensitivity	70%	81%
Specificity	98%	99%

**Table 3:** Summary of imaging methods used for detection of tumors in living mice.

Method	Physical basis	Reagents used	Spatial resolution	Reporter gene needed	Smallest detectable tumor (diameter)	Analysis time	Main advantages	Main disadvantages
T2W-MRI	Proton spin relaxation after radiowave emission	None	100 *μ*m	No	1 mm	3 hours/ mouse 30 hours/10 mice	High spatial resolution; Anatomical information; Gives tumor localization, size and morphology	Low throughput; Respiratory motion and high background make tumor detection in lungs challenging
FDG-PET	High-energy *γ* rays	^18^Fluoro-deoxy-glucose	2 mm	No	<1 mm	3 hours/ mouse 13 hours/ 10 mice	Detection of nonpalpable tumors; Quantifies tumor cell metabolism; Gives tumor localization	High background in some organs (brain, and bladder) prevents tumor detection in these regions
Biolumines-cence imaging	Visible light emitted during chemical reaction	D-luciferin substrate	1 to 10 mm dependant on tissue depth	Yes	<1 mm	1 hour/ mouse 2 hours/10 mice	Detection of nonpalpable tumors; Low background; Relative measure of tumor size; High throughput	Light emission dependant on 1/ tissue depth, 2/local availability of substrate reagents (luciferin, O_2_, and ATP)
Fluorescence imaging	Visible light emitted after fluorochrome excitation	None	1 to 10 mm dependant on tissue depth	Yes	2 mm	30 min/ mouse 1 hour/ 10 mice	High throughput	Light emission dependant on tissue depth; High background due to tissue autofluorescence
